# The Berlin-Brandenburg Air Study—A Methodological Study Paper of a Natural Experiment Investigating Health Effects Related to Changes in Airport-Related Exposures

**DOI:** 10.3389/ijph.2023.1606096

**Published:** 2023-11-17

**Authors:** Vanessa Soppa, Sarah Lucht, Katherine Ogurtsova, Anna Buschka, Mónica López-Vicente, Mònica Guxens, Kay Weinhold, Ulf Winkler, Alfred Wiedensohler, Andreas Held, Sabine Lüchtrath, Josef Cyrys, Simonas Kecorius, Petra Gastmeier, Miriam Wiese-Posselt, Barbara Hoffmann

**Affiliations:** ^1^ Institute of Occupational, Social and Environmental Medicine, Centre for Health and Society, Medical Faculty, Heinrich Heine University Düsseldorf, Düsseldorf, Germany; ^2^ Cardinal Health Real-World Evidence and Insights, Dublin, OH, United States; ^3^ ISGlobal, Barcelona, Spain; ^4^ Department of Experimental and Health Sciences, Universitat Pompeu Fabra, Barcelona, Spain; ^5^ Spanish Consortium for Research on Epidemiology and Public Health (CIBERESP), Instituto de Salud Carlos III, Madrid, Spain; ^6^ Department of Child and Adolescent Psychiatry/Psychology, Erasmus MC, University Medical Centre, Rotterdam, Netherlands; ^7^ Leibniz Institute for Tropospheric Research, Leipzig, Germany; ^8^ Environmental Chemistry and Air Research, Institute of Environmental Science and Technology, Technische Universität Berlin, Berlin, Germany; ^9^ Institute of Epidemiology, Helmholtz Zentrum München—German Research Center for Environmental Health, Neuherberg, Germany; ^10^ Institute of Hygiene and Environmental Medicine, Charité—Universitätsmedizin Berlin, Corporate Member of Freie Universität Berlin and Humboldt-Universität zu Berlin, Berlin, Germany

**Keywords:** air pollution, children’s environmental health, exposure assessment, methodological study, particulate matter

## Abstract

**Objectives:** This paper presents the study design of the Berlin-Brandenburg Air study (BEAR-study). We measure air quality in Berlin and Brandenburg before and after the relocation of aircraft (AC) traffic from Tegel (TXL) airport to the new Berlin-Brandenburg airport (BER) and investigate the association of AC-related ultrafine particles (UFP) with health outcomes in schoolchildren.

**Methods:** The BEAR-study is a natural experiment examining schoolchildren attending schools near TXL and BER airports, and in control areas (CA) away from both airports and associated air corridors. Each child undergoes repeated school-based health-examinations. Total particle number concentration (PNC) and meteorological parameters are continuously monitored. Submicrometer particle number size distribution, equivalent black carbon, and gas-phase pollutants are collected from long-term air quality monitoring stations. Daily source-specific UFP concentrations are modeled. We will analyze short-term effects of UFP on respiratory, cardiovascular, and neurocognitive outcomes, as well as medium and long-term effects on lung growth and cognitive development.

**Results:** We examined 1,070 children (as of 30 November 2022) from 16 schools in Berlin and Brandenburg.

**Conclusion:** The BEAR study increases the understanding of how AC-related UFP affect children’s health.

## Introduction

Ambient ultrafine particles (UFP) may pose a substantial risk to human health. However, epidemiological evidence for health effects of UFP in humans is still relatively scarce [1–3]. Due to their small size, UFP can penetrate deeply into the lungs, cross biological membranes, enter the bloodstream, and reach various organs, including the brain [1, 4]. Hypothesized health effects include local and systemic inflammation, oxidative stress, cardiovascular and respiratory problems, and potential impacts on the brain and metabolism [1, 2]. Short-term studies suggest associations between UFP and inflammation and cardiorespiratory changes, possibly independent of other pollutants [2].

Studies on aircraft (AC)-related UFP have shown that ambient UFP concentrations are strongly elevated near big airports, even 8–10 km downwind with measured UFP concentrations of 75,000 particles/cm^3^ and estimated particle number concentration (PNC) increases of 60,000 particles/cm^3^/minute within the first minutes after take-off at 380 m from the airport [5–12]. In the Netherlands near Amsterdam Schiphol airport, the contribution of air traffic to the annual average UFP was estimated to be about 5% of the total PNC and about 16% of 10–20 nm particles in Amsterdam (approx. 8 km away from the airport) [13, 14]. In addition to UFP generated directly from AC, airport-related road traffic also contributes to the UFP exposure of the population near large airports. Hudda et al. [6, 15] found that AC-UFP infiltrated indoors and resulted in significantly elevated PNC indoors.

Toxicological studies on the effect of AC-generated UFP on biological markers are rare. One recent study showed higher toxicity from AC-related particulate matter (PM) exposure than from PM from gasoline engines on human bronchial cells [16], whereas other studies have shown that AC emissions seem to have similar effects as standard diesel exhaust particles [17, 18].

Epidemiological studies on AC-related UFP and health outcomes are limited and yield mixed results [16–25]. Regarding long-term effects, an occupational study among airport workers in Copenhagen showed no associations between time spent working at the airport and incidence of ischemic heart disease or cerebrovascular disease [24]. Whereas a recent report on the health effects of long-term exposure to AC-UFP around Amsterdam Schiphol Airport showed that long-term exposure to AC-UFP may have an effect on the cardiovascular system for people living in the vicinity of Amsterdam Schiphol Airport [19]. Furthermore, there was a possible effect of the exposure of pregnant women to AC-UFP and the development of the unborn children [19]. These findings are supported by a recently published study from Wing et al. [23], which explored the relationship between joint exposure to airport-related noise, airport UFP, and road traffic-related air pollution (AP) on risk of preterm birth [22, 23].

Regarding short-term effects, Habre et al., 2018, observed associations of AC-UFP from the Los Angeles Airport (LAX) with inflammatory markers, whereas road traffic-related UFP were more strongly associated with lung function outcomes [26]. Further investigations on short-term AC-related PM exposure showed decreased lung function [20] and changes to the urinary metabolome in healthy volunteers [21]. Janssen et al. (2019) studied short-term UFP exposure near Schiphol Airport in Amsterdam, Netherlands, in both children and adults. They found that, on days with high exposure, children reported more respiratory complaints and medication use compared to adults. This effect was more pronounced in children who already had respiratory symptoms and were using medication [19].

Children, due to their incomplete lung and immune system development, are particularly vulnerable to air pollution [27]. Traffic-related air pollution has been linked to asthma and reduced lung function in children [28]. Additional research is needed, especially on the effects of AC-UFP and related emissions, particularly in children [29].

The BEAR study leverages an airport transition to investigate AC-UFP and airport-related exposures’ effects on children’s health. With the closure of Berlin Tegel Airport (TXL), in the north of Berlin, and the opening of Berlin-Brandenburg Airport (BER), on the southern boarder of Berlin, in November 2020, the study seizes a unique opportunity for a natural experiment. It focuses on understanding short-, medium-, and long-term health effects of airport-related exposures, including cardiovascular, respiratory, and neurocognitive health in children. This study aims to improve data for deriving air quality standards. Here, we detail the study design, recruitment, and assessment procedures of air pollution and health within the BEAR study.

## Methods

### Study Design

The BEAR study is a prospective cohort study on children, using a natural experiment of the relocation of air traffic in the city of Berlin.

Prior to October 2020, Berlin had two operating airports, TLX and Berlin Schönefeld Airport (SFX) with about 2 million (TXL) and 1 million (SFX) passengers per month. A third airport was under construction directly south of SFX (Berlin-Brandenburg Airport, BER) (Supplementary Figure S1). SFX operated as an independent airport until the end of October 2020. The airport’s check-in building was integrated into BER as an additional terminal, but has not been used since February 2021. The relocation of air traffic from TLX to BER in November 2020 implied a total loss of air traffic at TLX and thus a drastic reduction of airport-related emissions for children living near TLX. Simultaneously, through the commissioning of BER, a sudden and strong increase of air traffic and of airport-related emissions at SFX and BER, was expected, subjecting the children living near SFX/BER to substantial increases in aircraft-related exposures. Children living in areas away from all airports [control areas (CA)] are assumed to be not affected by the air traffic relocation.

**FIGURE 1:**
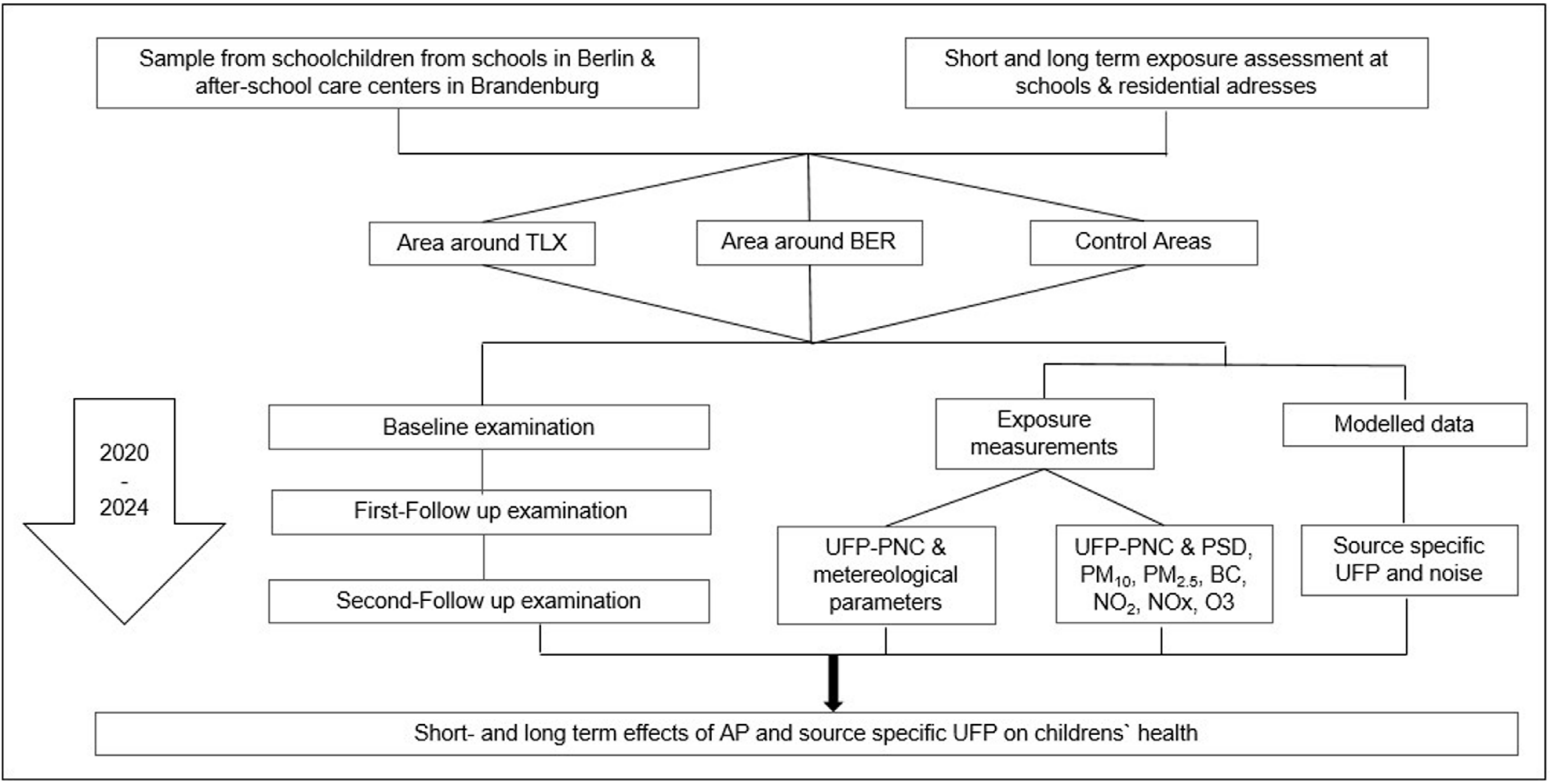
Study design of the Berlin-Brandenburg Air Study (Berlin-Brandenburg Air Study, Germany 2020–2024).

We recruited children attending elementary schools and their affiliated after-school centers in the city of Berlin and in the federal state of Brandenburg: near TXL, near BER, and from CA in Berlin (Supplementary Figure S1). Parallel to the repeated health examinations of the children, we measure the outdoor UFP concentration at the participating schools. AP is measured continuously at several measuring stations in Berlin and Brandenburg near TLX, SFX and BER as well as in CA. Modelled source-specific UFP and other air-pollutants at the schools and home addresses of participating children will be available for long-term exposure pre- and post-relocation of Berlin AC traffic (Figure 1).

Health examinations started in January 2020. Follow-up examinations are ongoing and are expected to end in May 2024. The BEAR study has been approved by the local ethics committee at Heinrich-Heine-University of Düsseldorf (protocol number: 2019-695). For all children, a parent or legal guardian provided informed consent. From a list of all primary schools in Berlin, we prioritized eligible schools according to location (Supplementary Figure S1), comparable SES of the neighborhoods, local road traffic exposure and exposure to industrial sources of AP and noise.

For prioritization, we used the sociodemographic atlas of Berlin [30] and the existing noise and road traffic maps of Berlin [31]. Initially, we planned to examine altogether 550 schoolchildren aged 7–12 years.

### Health Examinations

Health examinations started in January 2020 in Berlin and December 2020 in Brandenburg, with baseline examinations completed by December 2022. Parallel to baseline examinations, the follow-up examinations started in August 2021 and are still ongoing. These examinations were integrated into the regular school day in Berlin and after-school day-care in Brandenburg, accompanied by an educational module on healthy living performed by the study staff. To ensure consistency, study staff underwent regular training, certification, recertification, and process monitoring. All tests adhered to standard operating procedures in line with international and national standards. Parents who consented to participate completed a comprehensive questionnaire covering their child’s health, COVID-19 history, parental demographics, and family lifestyle factors (in German Supplementary Baseline Questionnaire parents).

At each examination, acute physical illnesses and medication use were assessed with an examiner-conducted questionnaire (in German Supplementary Acute Health Questionnaire).

Quality of life (QoL) was evaluated using the KIND-L (“Revidierter Fragebogen für KINDer und Jugendliche zur Erfassung der gesundheitsbezogenen Lebensqualität”) questionnaire for children through interviews (in German Supplementary KINDL Questionnaire).

We measured height and weight without shoes and in light clothing with a fixed ruler and a digital scale, respectively. For blood pressure measurements, children were placed in an upright sitting position with their arms placed on the table in front of them. The blood pressure cuff was placed on the arm used for writing. Care was taken to ensure that clothing did not constrict the arm. After resting for 5 minutes, systolic and diastolic blood pressure measurements were taken in triplicate with 2 minutes rest in between measurements using a digital oscillometric device (ProBP 2000, Welch Allyn).

Exhaled nitric oxide (FeNO) tests (NIOX VERO, Circassia) were conducted, with children attempting the 10 s version first and switching to the 6 s version if necessary. High FeNO values (>35 ppb) were confirmed in a second test for those children. FeNO tests were conducted before spirometry in order to avoid biasing nitric oxide values towards the null.

Spirometry tests were performed in accordance with American Thorax Society (ATS) and European Respiratory Society (ERS) guidelines [32] using the EasyOne spirometer (NDD Medical Technologies). Children underwent an initial eligibility screening with exclusion criteria such as current acute infections, recent chest or torso surgery within the past 3 months, recent eye surgery within the past month, or the use of tuberculosis medication. Children with asthma taking medication were allowed to participate if their last use was sufficiently long before the lung function test, ranging from up to 4 h (short-acting ß2 mimetics like salbutamol) to 5 days (leukotriene antagonist like montelukast).

During the lung function test, children were seated upright with their feet on the ground, maintaining a proper distance from the table with the computer screen. They watched an instructional video demonstrating full spirometry with forced maximal exhalation and received verbal instructions for the maneuver. They were required to perform two consecutive maneuvers, with only the second one analyzed. Valid maneuvers met the following criteria: back extrapolated volume (BEV) <0.15 L, time to peak flow (PEFT) <0.12 s, and either forced expiratory time (FET) >3 s or end of test volume (EOTV) <0.04 L. Valid maneuvers were assessed for reproducibility, and the lung function test ended after obtaining three valid and reproducible maneuvers or reaching a maximum of eight maneuvers. If fewer than two valid and reproducible maneuvers were achieved, a repeat examination was scheduled.

To assess cognitive function, children completed the standardized N-back and Attentional Network Task (ANT) Test. The N-back test is a well-established working memory assessment where participants determine if the current color matches the one shown n colors back [33–35]. It was conducted for 1-, 2-, and 3-back conditions, generating scores based on correct and incorrect answers. The ANT test evaluates various forms of attention (alerting, orienting, and conflict) by presenting an image of five fishes in a line [36]. Children click the mouse button corresponding to the middle fish’s direction, with reaction times and accuracy recorded and calculated into scores [36]. Both tests were programmed in e-prime [37] and administered at separate tables with headphones and privacy screens to ensure a quiet environment. Children received instructions and a test run to ensure task comprehension.

We plan to examine each child at least three times until spring 2024. Should children move schools or no longer choose to participate, we will recruit additional participants within the schools already participating in the study.

### Air Pollution Assessment

Air pollutant concentrations near Berlin airports and in the CA have been assessed at several monitoring sites mostly since 2019. A detailed description of the measurement stations is given in Table 1 and the location in the Supplementary Figure S1. All data collected at the measurement stations will be made available for the BEAR project.

**TABLE 1:** Description of fixed-site measurement sites providing data for the Berlin-Brandenburg Air Study (Berlin-Brandenburg Air Study, Germany 2020–2024).

Site	Location	Time PerioD	Operated by	Measurement program
1 RKD	Reinickendorf, 4 km east of TXL	July 2020–June 2021	BEAR Consortium (TROPOS)	PNC (7 < D_p_), PSD (10 < D_p_ < 800 nm), eBC, PM_10_, PM_2.5_, NO_x_, NO_2_, O_3_, CO
2 TUB	Urban background (rooftop), 5.7 km southeast of TXL	March 2017–March 2022	Technische Universität Braunschweig	PSD (6 < D_p_ < 560 nm)
3 HUB	Urban background (rooftop), 6.5 km north of SFX/BER	January 2020–ongoing	Humboldt University Berlin	PNC (7 < D_p_), PM_10_, PM_2.5_, PM_1_, meteorology
4 BDF	Bohnsdorf, 6 km north-east of the BER terminal	PSD measurements since March 2018–ongoing	Airport Berlin Brandenburg GmbH	PSD (5 < D_p_ < 1,094 nm), PM_10_, PM_2.5_, NO_2_, NO_x_, O_3_, CO, meteorology
5 SFX	SFX air field	PSD measurements since October 2016–ongoing	Airport Berlin Brandenburg GmbH	PSD (5 < D_p_ < 1,094 nm), PM_10_, PM_2.5_, BC, NO_2_, NO_x_, O_3_, CO, meteorology
6 UFB	Blankenfelde-Mahlow, 5 km west of the BER terminal	June 2021–October 2022	ULTRAFLEB (TROPOS)	PNC (7 < D_p)_, PSD (10 < D_p_ < 800 nm)
7 BFM	Blankenfelde-Mahlow, 5 km west of the BER terminal	May 2020–ongoing	Network monitoring station of the Brandenburg Environment Agency	PNC (7 < D_p_), eBC, PM_10_, PM_2.5_, NO_x_, NO_2_, O_3_, CO

The monitoring of air quality began in July 2020 at a fixed-site monitoring station in the downwind area of the old airport TXL in order to assess the change in pollutant concentrations in the direct vicinity of the TXL school sites Berlin-Reinickendorf (RKD). In June 2021, this fixed monitoring station was moved to Blankenfelde-Mahlow, near the new airport BER. This measurement station was operated by the Leibniz Institute for Tropospheric Research (TROPOS) by October 2022 in the framework of the research project “Ultrafeinstaubbelastung durch Flughäfen in Berlin” (ULTRAFLEB), a BEAR-associated 3 years research project funded through the German Federal Environment Agency. Near the new airport BER, two additional fixed measurement stations including highly time-resolved PNC measurements (GRIMM 5420) were already present. The airport company “Flughafen Berlin Brandenburg” (FBB) operates both monitoring stations. The first one is located directly adjacent to the airfield (site SFX) and a second one in Berlin-Bohnsdorf (BDF) about 5 km downwind of SFX/BER since October 2016.

In order to estimate UFP concentrations at the recruited schools, a condensation particle counter (CPC; EDM465, Grimm Aerosol Technik, model EDM465) was placed to school sites concurrently with the baseline health examinations. This device measures total PNC in the size range from 7 nm to 1 µm utilizing pre-impactor. This measurement campaign started in January 2020 and is still ongoing.

To distinguish between UFP emissions from aircraft-related sources and other PNC sources like road traffic and wood combustion, we employ various strategies. Initially, we utilize backward air mass trajectories to assess airport influence at school locations. By defining sectors where the airport is downwind, we can estimate airport-related particle signals in the CPC measurements, accounting for nearby roads and highways. Further analysis relies on particle size distribution (PDS) data and additional air pollutant measurements gathered from monitoring stations in the vicinity of the airports (Table 1). We conduct correlation analyses between PNC at schools and monitoring sites. After determining how those stations represent the particle number concentrations at schools (work ongoing in separate publication), long-term high quality data will be used to run source apportionment by use of positive matrix factorization (PMF) method (following methodology by, e.g., Tremper et al., 2022) [38], segregating between different pollution sources, including airport and traffic. Finally, the results of the source apportionment will be transferred to the school locations.

It’s worth noting that our study quantifies airport activity-induced concentration increases in total PNC, which we convert to UFP PNC based on PDS measurements, Although several studies do refer to total PNC as UFP [12, 39–41]. To validate our approach, we examine particles with a mobility diameter of 30 nm, indicative of airport-related activities [38].

Additionally, ULTRAFLEB provides BEAR with modeled source-specific UFP and air pollutant data for long-term exposure assessments pre- and post-relocation of Berlin AC traffic (see Supplementary Figure S1). This modeling covers Berlin and Brandenburg, including residential addresses of participating children and schools, at a spatial resolution of 50 m*50 m. We generate exposure estimates for PM_10_, PM_2.5_, NO_2_, black carbon, and UFP from 2019 to 2023, validating them against school site, monitoring station, and mobile campaign data.

### Planned Analyses

We plan to analyze short-, medium- and long-term effects of total and source-specific UFP. Short-term periods refer to hourly and daily, as medium- and long term periods refer to monthly and yearly effects of air pollution measured and modelled over the appropriate time at children`s schools and residential addresses.

To identify the appropriate confounders for each analysis, we will employ a directed acyclic graph (DAG) approach. This method allows us to identify the minimal sufficient adjustment sets needed to establish the association between UFP exposure and the outcome parameters. Possible confounders may include socio-economic status (SES) of the area or parents, history of chronic diseases (e.g., asthma), history of attention deficit disorder/attention deficit hyperactivity disorder, season, and other relevant factors.

### Short-Term Analyses

We will initially assess short-term changes in various health factors, including respiratory symptoms, lung function, cognitive function, blood pressure, and quality of life. We’ll use generalized linear mixed models, considering exposure to air traffic, location (TXL, BER, CA), and examination time as variables. This analysis will involve all collected data across all groups. For source-specific (AC-UFP) exposure, we will include other airport-related factors like road traffic noise, air pollution (AP), and changes in socioeconomic-status (SES) in sub-analyses. We will also explore different exposure time windows based on existing literature. To ensure accuracy, we will adjust our models for factors such as age, gender, weather conditions, background air pollution, NDVI (normalized difference vegetation index), and relevant covariates that could influence both exposure and outcomes (confounding variables).

### Long-Term Analyses

The primary outcomes of long-term health effects are lung growth and cognitive development. We will consider background air pollution (AP) exposure through a designated control region. The central hypothesis of the BEAR Study is that reducing AC-UFP and other airport-related exposures near TXL will improve lung and cognitive function, while an increase in these exposures near SFX/BER will reduce lung and cognitive function. To thoroughly investigate these effects, we will analyze changes in pollutant levels downwind of the airports and in CA, changes in exposure for schoolchildren near TXL, SFX/BER, and in CA, and changes in the children’s health outcomes over time.

Our analysis will involve linear mixed-effects models, assessing lung capacity (FVC and FEV_1_) from up to three visits, as well as working memory and attention. We will include a random participant intercept for each child and account for temporal trends in exposure and outcomes over the 4 years study period, using the examination date as a variable. We will also consider seasonal, meteorological, and other variables that could predict the outcomes. Similar to the short-term analysis, the models will be adjusted for age, gender, weather conditions, background air pollution, NDVI, and potential confounding factors, which may vary depending on the specific outcome under study. We will particularly address noise-related confounding by incorporating changes in exposure to different noise sources based on modeled noise levels at residential addresses.

### Source-Specific Analyses

In addition to these analyses we will conduct a sub-analysis focusing solely on individuals exposed to airport-related UFP to assess the source-specific short-term effects more precisely. This sub-analysis will allow us to gain deeper insights into the specific impacts of airport-related UFP exposure on the outcomes under study. In contrast, for the long-term analysis, we will utilize a binary exposure variable that distinguishes between two groups: those living near the airport and the control group. This approach will allow us to examine and compare the differences in lung growth and cognitive development over time between these two distinct groups.

### Mediation Analysis

Potential mediators of the total effect of the intervention on health outcomes in our analysis are changes in AC-related UFP or other AC-related air pollutants, road traffic-related UFP or other road traffic-related air pollutants, AC-noise, road traffic noise, and area-level SES. Outcomes will be short-term changes in lung function, lung inflammation, and cardiovascular health, as well as medium- and long-term changes in lung growth and cognitive development. We will conduct mediation analyses according to the principles and methods as outlined by VanderWeele [42]. We will also conduct sensitivity analyses evaluating whether the exposure and mediators may interact in their effect on the outcome.

### Sample Size Calculation

We calculated the detectable effect size for our study based on dynamic lung function volumes (FEV_1_, FVC) as the outcome. We will assess 1,070 children, resulting in a total of 3,210 observations (three observations per child). With an average FEV_1_ of 1.6 L (SD 0.27 L) in elementary school children in Germany [43], a 5% alpha-level, and a sample size of 503 children, we have at least 80% power to detect a 0.030 L reduction in FEV_1_. This change is similar to what’s observed after a 2 h exposure of participants with mild to moderate asthma per 10,000 particles/cm^3^ [44] and smaller than daily changes in ambient PM AP on lung function in children [45, 46]. With a sample size of 856 children, we can detect a 0.015 L reduction in FEV_1_ (alpha-level of 5% and 80% power), allowing for a 20% loss of participants.

For the analysis of short-term effects on FeNO, we estimate that with a mean FeNO level of 11 ppb (SD 1.1 ppb), we can detect a moderate effect (0.5 ppb decrease) with 232 measurements, using a 5% alpha level and 90% power, with an allocation ratio of 2:1.

Regarding the analysis of lung growth trajectories and cognitive development, we can detect small to moderate differences with an alpha-level of 5% and 80% power in a sample of 282 children.

## Results

We recruited 1,070 children (as of 30 November 2022) from 16 schools in Berlin and Brandenburg, with the number of participating children varying from school to school (Table 2; Figure 3). The children listed here had already completed at least one and a maximum of three health examinations.

**TABLE 2:** Participating schools in Berlin and Brandenburg and completed baseline examinations (as of 30 November 2022) (Berlin-Brandenburg Air Study, Germany 2020–2024).

Study area	Elementary school (district of Berlin/municipality of Brandenburg)	N included
Control Area (CA)	CA-1 (Berlin-Johannisthal)	93
	CA-2 (Berlin-Oberschöneweide)	64
	CA-3 (Berlin-Neukölln)	67
TLX	TLX-1 (Berlin-Reinickendorf)	90
	TLX-2 (Berlin-Reinickendorf)	90
	TLX-3 (Berlin-Reinickendorf)	111
SFX/BER	BER-1 (Berlin-Bohnsdorf)	146
	BER-2 (Berlin-Altglienicke)	64
	BER-3 (Schulzendorf, Brandenburg)	67
	BER-4 (Eichwalde, Brandenburg)	54
	BER-5 (Schönefeld, Brandenburg)	52
	BER-6 (Schönefeld, Brandenburg)	42
	BER-7 (Blankenfelde-Mahlow, Brandenburg)	33
	BER-8 (Blankenfelde-Mahlow, Brandenburg)	32
	BER-9 (Blankenfelde-Mahlow, Brandenburg)	16
	BER-10 (Blankenfelde-Mahlow, Brandenburg)	49
Total		1,070

### Design Adaptations Due to the COVID-19 Pandemic

**FIGURE 2:**
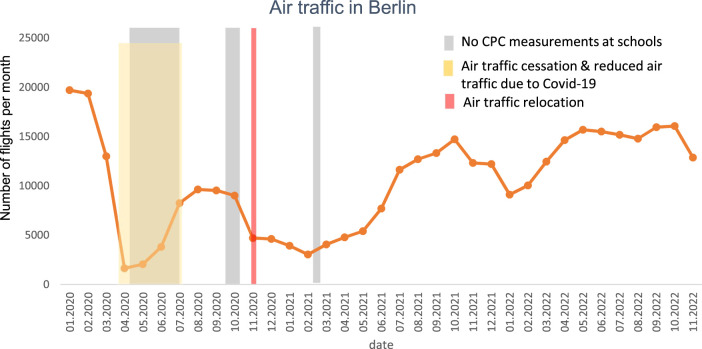
Number of flights (departure and arrival) at Tegel Airport, Schönefeld Airport and Berlin-Brandenburg Airport as well as air traffic cessation and relocation of air traffic in Berlin and Brandenburg [47, 48] (Berlin-Brandenburg Air Study, Germany 2020–2024).

As in many studies, the appearance of COVID-19 in late 2019 and the subsequent global pandemic have significantly impacted several aspects of this study. The natural experiment involving an abrupt closing of TXL Airport and a quick takeover of all air traffic by BER did not occur due to the large-scale cessation of air traffic starting in March 2020 at TLX and SFX. In summer 2020 reduced air traffic resumed TLX, and in November 2020 air traffic relocated from TLX to BER but still with a reduced volume (Figure 2).

**FIGURE 3:**
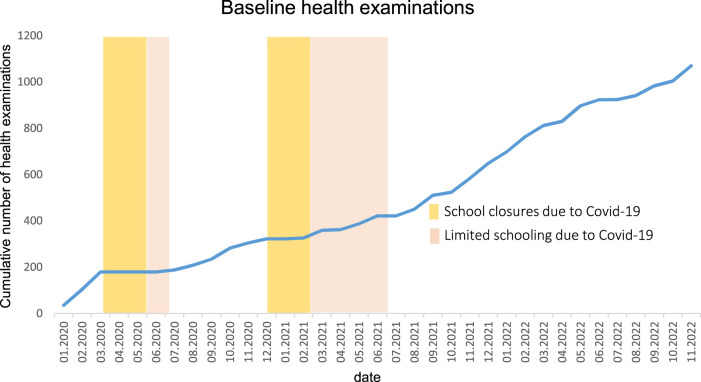
Cumulative number of baseline health examinations with dates of school closure and limited schooling in Berlin and Brandenburg due to COVID-19 pandemic (Berlin-Brandenburg Air Study, Germany 2020–2024).

With the gradual increase in air traffic at BER we conducted more ‘low exposure’ measurements in children attending schools in that area and had fewer measurements available under high exposure conditions. Additionally, because of intermittent school closures due to high local COVID-19 incidence rates (Figure 3), recruitment and examination of children were more difficult than expected. As a contingency measure, we decided to include additional schools near BER Airport in Brandenburg and to extend our observation period to at least 4 years. With the addition of new study areas in Brandenburg, we increased the number of participants to more than 1,000 schoolchildren (Table 2).

## Discussion

The BEAR study represents an important step forward in the attempt to understand how AC-UFP as well as other AC-related emissions may influence the short- and long-term health of populations living near airports.

A particular strength of the BEAR study is the design of a natural experiment, as we used the abrupt relocation of Berlin air traffic to determine the association of short- and long-term exposure to AC-UFP and health effects of children. We chose to focus in our study on elementary schoolchildren for several reasons: In Berlin and Brandenburg, elementary schoolchildren usually attend the school that is closest to their residence and often in walking distance from their home. Moreover, a large fraction of elementary school children stay on the school grounds for afternoon care. Therefore, exposure measured at the school can be used as a surrogate for daily exposure. In addition, we assume that other exposures often encountered in adults (active smoking, spending a lot of time in high traffic, occupational exposures), which can all lead to individual exposure estimation error, do not apply for children. Finally, children are usually very physically active, have high minute ventilation in relation to their body size, leading to a high intake of air pollutants, and are particularly susceptible due to their growing and developing organs [49].

Another strength is the comprehensive air quality measurement campaign in Berlin and Brandenburg. We will be able to assess concentration changes in size-fractioned and source-specific UFP, other air pollutants (PM_10_, PM_2.5_, BC, NO_2_, NOx, O_3_), other airport-related exposures (i.e., aircraft noise, road traffic noise) due to the relocation of air traffic from TXL to BER for schoolchildren in the vicinity of both airports and in CA not impacted by AC-associated air pollutants. In combination with the comprehensive health examinations on schoolchildren, we will be able to detect health effects related to changes in airport-related exposures. The BEAR study is therefore well-situated to provide insight into how AC-related exposures affect children’s quality of life, lung and cognitive development and cardiovascular health, outcomes which have lifelong consequences [29, 50, 51].

At present, there are no regulatory standards governing UFP levels in ambient air. Despite growing evidence showing that exposure to these small particles has health consequences [1, 2, 4, 50], the World Health Organization (WHO) did not considered the evidence strong enough to derive guidelines for UFP in its most recent Air Quality Guidelines [53]. Similarly, regulatory bodies have deemed the evidence insufficient to justify the introduction of air quality standards for UFP [53–55]. Nevertheless, in the revised WHO guidelines 2021, good practice statements were formulated in order to address concerns about the health and environmental effects of UFP [53]. According to those statements, PNC < 1,000 particles/cm^3^ (24 h mean) can be considered as low and potentially harmless, whereas PNC > 10,000 particles/cm^3^ (24 h mean) or 20,000 particles/cm^3^ (1 h) are classified as high. With the knowledge arising from the BEAR study, particularly from a population considered highly vulnerable to AP, we hope to further efforts by scientists to introduce UFP standards that will ultimately lead to improved health for many.

Through AP monitoring and modeling as well as regular health examinations among schoolchildren in Berlin and Brandenburg, the ongoing BEAR study represents a unique opportunity to advance our scientific knowledge about how AC-specific UFPs influence childhood development and health.

## Figures and Tables

**Table:** 

**AC**	aircraft
**AC-UFP**	aircraft related ultrafine particles
**AP**	air pollution
**ATS**	American Thoracic Society
**BEAR**	Berlin-Brandenburg Air Study
**BER**	Berlin-Brandenburg Airport
**BC**	Black Carbon
**BDF**	Bohnsdorf
**BFM**	Blankenfelde-Mahlow
**CA**	Control Area
**CPC**	condensation particle counter
**ERS**	European Respiratory Society
**FBB**	Flughafen Berlin Brandenburg GmbH
**FEV**_**1**_	forced expiratory volume in 1 s
**FVC**	forced vital capacity
**HEI**	Health Effects Institute
**HUB**	Humboldt Universität zu Berlin
**KIND-L**	Revidierter Fragebogen für KINDer und Jugendliche zur Erfassung der gesundheitsbezogenen ebensqualität
**LAX**	Los Angeles International Airport
**NDVI**	normalized difference vegetation index
**NO**_**2**_	nitrogen dioxide
**PM**	particulate matter
**PM**_**0.1**_	particulate matter with an aerodynamic diameter <0.1 µm
**PM**_**2.5**_	particulate matter with an aerodynamic diameter <2.5 µm
**PM**_**10**_	particulate matter with an aerodynamic diameter <10 µm
**PNC**	particle number concentration
**PSD**	particle size distribution
**RKD**	Reinickendorf
**SES**	socioeconomic-status
**SFX**	Berlin-Schönefeld Airport
**TROPOS**	Leibniz Institute for Tropospheric Research
**TUB**	Technische Universität Braunschweig
**TXL**	Berlin-Tegel Airport
**UFP**	ultrafine particles
**UFB**	ULTRAFLEB, Ultrafeinstaubbelastung durch Flughäfen in Berlin
