# Supplementary material for: The Berlin-Brandenburg Air Study—A Methodological Study Paper of a Natural Experiment Investigating Health Effects Related to Changes in Airport-Related Exposures
Source: Int J Public Health. 2023 Nov 17;68:1606096. doi: 10.3389/ijph.2023.1606096 (PMC10689260; doi:10.3389/ijph.2023.1606096)
Supplement: Supplementary file 1 [file DataSheet2.pdf]

Fragebogen für Eltern

Abkürzung Schule : |___|___|___| *(wird vom Studienpersonal ausgefüllt)*

ID-Nummer : |___|___|___|___|___| *(wird vom Studienpersonal ausgefüllt)*

Name des Kindes : ____

(wird vom Studienpersonal ausgefüllt und nach Erhalt geschwärzt)

Liebe Eltern,

mit diesem Fragebogen möchten wir mehr über Ihr Kind, das Teil unserer Studie ist, und dessen Umgebung erfahren. Die nachfolgenden Fragen sind wichtig für unsere wissenschaftliche Auswertung. Wir möchten Sie bitten, den Fragebogen nach Möglichkeit vollständig und **innerhalb von 2-3 Wochen** auszufüllen und ihrem Kind wieder mitzugeben, damit wir diesen in der Schule/im Hort einsammeln können.

Ihre Angaben werden gemäß den Datenschutzbestimmungen vertraulich und anonymisiert behandelt und ausschließlich zu wissenschaftlichen Zwecken ausgewertet.

Wir möchten Ihnen schon jetzt herzlich für Ihre Angaben danken, die entscheidend zum Erfolg dieser Untersuchung beitragen werden.

Bei Rückfragen wenden Sie sich gerne an unsere Studienbetreuerin, Frau Kerstin Theisen:
Email: kerstin.theisen@charite.de
Telefon: +49 (0) 173 – 860 089 1

Wie der Fragebogen auszufüllen ist:

Benutzen Sie einen schwarzen oder blauen Kugelschreiber.
Kreuzen Sie bitte die jeweils zutreffende Antwort im dafür vorgesehenen Kästchen an.
Machen Sie das Kreuz genau in das Kästchen und nicht darüber: ☒
Wählen Sie immer nur diejenige Antwort, die am besten zutrifft. Machen Sie bitte keine Kreuze zwischen den Kästchen**!**
Auf die meisten Fragen geben Sie bitte nur eine Antwort. Wenn mehrere Antworten auf eine Frage angekreuzt werden können, werden Sie darauf hingewiesen, mit dem Vermerk:
„❖ Mehrfachnennungen möglich"

Bitte handschriftliche Angaben in gut lesbarer Druckschrift notieren

Beispiele: |___|_6_| [Jahre]; Name Medikament: ___Salbutamol___

Falls der Platz nicht ausreichen sollte, bitten wir Sie, Ihre Antworten mit Angabe der Frage auf einem Extrablatt aufzuführen und diesem Fragebogen beizulegen.
Falls eine Frage übersprungen werden kann, werden Sie bei entsprechender Antwort darauf hingewiesen und können dem grauen Pfeil direkt zur nächsten Frage folgen:

… *„→ Gehen Sie bitte weiter zu Frage …"*

Start

S1 Datum des Ausfüllens

|___|___|.|___|___|.|___|___|___|___|
[TT.MM.JJJJ]

S2 Uhrzeit des Ausfüllens

|___|___|.|___|___| [hh:mm]

S3 Wer füllt den Fragebogen aus? ❖ *Mehrfachnennungen möglich*

☐$_1$ Mutter ☐$_1$ Vater ☐$_1$ Andere Person

S4 In welchem Land wurde die leibliche Mutter des Kindes geboren?

☐$_1$ Deutschland ☐$_2$ In einem anderen Land und zwar:

____
Geburtsland

S5 In welchem Land wurde der leibliche Vater des Kindes geboren?

☐$_1$ Deutschland ☐$_2$ In einem anderen Land und zwar:

____
Geburtsland

1
2
frühere Version

Angaben zum Kind

K1 Geburtsdatum

|___|___|.|___|___|.|___|___|___|___|
[TT.MM.JJJJ]

K2 In welchem Land wurde Ihr Kind geboren?
❖ *Bitte verwenden Sie die heutige Staatsbezeichnung!*

☐$_1$ Deutschland ☐$_2$ In einem anderen Land und zwar:

____
Geburtsland

K3 Geschlecht

☐$_1$ Junge ☐$_2$ Mädchen

ID-Nummer: |___|___|___|___|___| [wird vom Studienpersonal eingetragen]

K4 Aktuelle Wohnadresse des Kindes ❖ *Bei mehreren Adressen bitte alle angeben*

1. Wohnadresse

Straße, Hausnummer

|____|

PLZ, Ort |__|__|__|__|__| |______________________________|

Wie viele Tage in der Woche: |___|

2. Wohnadresse

Straße, Hausnummer

|____|

PLZ, Ort |__|__|__|__|__| |______________________________|

Wie viele Tage in der Woche: |___|

❖ *Bei weiteren Adressen bitte auf der Rückseite notieren*

2 | 3 *frühere Version*

K5 Sind Sie in den vergangenen 4 Jahren umgezogen?

☐1 Ja ☐0 Nein ➔ *Gehen Sie bitte weiter zu Frage* ***K6 auf der Seite 5***

K5.1 Wenn „Ja", geben Sie bitte das Datum/die Daten des Umzugs und die alte Adresse an

1. Umzug

|___|___|.|___|___|.|___|___|___|___| [TT.MM.JJJJ]

Alte Anschrift:

Straße, Hausnummer

|____|

PLZ, Ort |__|__|__|__|__| |______________________________|

2. Umzug

|___|___|.|___|___|.|___|___|___|___| [TT.MM.JJJJ]

Alte Anschrift:

Straße, Hausnummer

|____|

PLZ, Ort |__|__|__|__|__| |______________________________|

❖ *Bei weiteren Adressen bitte auf der Rückseite notieren*

CHARITÉ

hhu Heinrich Heine Universität Düsseldorf

**K6**	Wie kommt Ihr Kind meistens zur Schule?

☐$_1$ Zu Fuß
☐$_2$ Mit dem Fahrrad oder Roller
☐$_3$ Mit Bus/Bahn
☐$_4$ Mit dem Auto

3 | 4 *frühere Version*

Medizinische Anamnese

**A1**	Hatte Ihr Kind irgendwann einmal beim Atmen pfeifende oder keuchende Geräusche im Brustkorb?	
	☐$_1$ Ja	☐$_0$ Nein → *Gehen Sie bitte weiter zu Frage **A2***
A1.1	Hatte Ihr Kind in den letzten 12 Monaten beim Atmen pfeifende oder keuchende Geräusche im Brustkorb?	☐$_1$ Ja<br>☐$_0$ Nein → *Gehen Sie bitte weiter zu Frage **A2***
A1.2	Wie viele Anfälle von pfeifender oder keuchender Atmung hatte Ihr Kind in den letzten 12 Monaten?	☐$_1$ Keinen Anfall<br>☐$_2$ 1 - 3 Anfälle<br>☐$_3$ 4 - 12 Anfälle<br>☐$_4$ mehr als 12 Anfälle
A1.3	War die pfeifende oder keuchende Atmung in den letzten 12 Monaten jemals so stark, dass Ihr Kind beim Reden schon nach 1-2 Worten wieder Luft holen musste?	☐$_1$ Ja<br>☐$_0$ Nein
A1.4	Hatte Ihr Kind in den letzten 12 Monaten pfeifende, keuchende Atemgeräusche im Brustkorb während oder nach körperlicher Anstrengung?	☐$_1$ Ja<br>☐$_0$ Nein
A1.5	Wie oft ist Ihr Kind in Durchschnitt in den letzten 12 Monaten wegen pfeifender oder keuchender Atmung aufgewacht?	☐$_1$ Nie deswegen aufgewacht<br>☐$_2$ Weniger als 1 Nacht pro Woche<br>☐$_3$ 1 oder mehrere Nächte pro Woche
**A2**	Hatte ihr Kind irgendwann einmal Asthma?	
	☐$_1$ Ja	☐$_0$ Nein → *Gehen Sie bitte weiter zu Frage **A3***
A2.1	In welchem Alter des Kindes trat das Asthma zum ersten Mal auf?	|___|___| [Jahre]
A2.2	Besteht die Erkrankung weiterhin?<br>☐$_0$ Nein	☐$_1$ Ja → *Gehen Sie bitte weiter zu Frage **A2.3***
A2.2.1	Seit welchem Alter ist Ihr Kind beschwerdefrei?<br>|___|___| [Jahre]	
A2.3	Ist Ihr Kind in den letzten 12 Monaten wegen Asthma behandelt worden	
	☐$_1$ Ja	☐$_0$ Nein → *Gehen Sie bitte weiter zu Frage **A2.4***

4 | 5 *frühere Version*

A2.3.1 Geben Sie bitte die Medikamente, auch Inhalationsmittel, an, mit denen es behandelt wurde.
❖ *Bei weiteren Medikamenten bitte auf einem Extrablatt auflisten*

1. Medikament

Name: ______________________________

mg: ______________________________

Wie häufig (falls bekannt):

____

2. Medikament

Name: ______________________________

mg: ______________________________

Wie häufig (falls bekannt):

____

3. Medikament

Name: ______________________________

mg: ______________________________

Wie häufig (falls bekannt):

____

A2.4 Ist Ihr Kind jemals wegen Asthma mit sogenannten alternativen Methoden (Homöopathie, Akupunktur, Bioresonanz) behandelt worden?

☐$_1$ Ja ☐$_0$ Nein → *Gehen Sie bitte weiter zu Frage* **A3**

A2.4.1 Bitte geben Sie die Art der Behandlung an

____

____

A2.4.2 Wie alt war Ihr Kind?

|___|___| [Jahre]

A3 Hatte Ihr Kind <u>in den letzten 12 Monaten</u> jemals nachts einen trockenen Reizhusten, obwohl es keine Erkältung oder Bronchitis hatte?

☐$_1$ Ja ☐$_0$ Nein

A4 Hatte Ihr Kind <u>irgendwann einmal</u> Niesanfälle oder eine laufende, verstopfte oder juckende Nase, obwohl es nicht erkältet war?

☐$_1$ Ja ☐$_0$ Nein → *Gehen Sie bitte weiter zu Frage* **A5**

A4.1 Hatte Ihr Kind <u>in den letzten 12 Monaten</u> Niesanfälle oder eine laufende, verstopfte oder juckende Nase, obwohl es nicht erkältet war?

☐$_1$ Ja
☐$_0$ Nein → *Gehen Sie bitte weiter zu Frage* **A5**

5/6 *frühere Version*

A4.2	Hatte Ihr Kind <u>in den letzten 12 Monaten</u> gleichzeitig mit diesen Nasenbeschwerden auch juckende oder tränende Augen?	☐ 1 Ja<br>☐ 0 Nein
A4.3	Wann in den letzten 12 Monaten traten die Nasenbeschwerden auf?<br>❖ *Mehrfachnennungen möglich*	☐ Januar ☐ Mai ☐ September<br>☐ Februar ☐ Juni ☐ Oktober<br>☐ März ☐ Juli ☐ November<br>☐ April ☐ August ☐ Dezember
A4.4	Wie stark war Ihr Kind <u>in den letzten 12 Monaten</u> durch die Nasenbeschwerden in seinen Aktivitäten eingeschränkt?	☐ 1 Gar nicht ☐ 3 Mittelstark<br>☐ 2 Wenig ☐ 4 Stark

6/7 *frühere Version*

A5 Hatte Ihr Kind <u>irgendwann einmal</u> Heuschnupfen?

☐ 1 Ja

☐ 0 Nein → *Gehen Sie bitte weiter zu Frage* ***A6***

A5.1 Ist Ihr Kind <u>in den letzten 12 Monaten</u> wegen Heuschnupfen behandelt worden?

☐ 1 Ja

☐ 0 Nein → *Gehen Sie bitte weiter zu Frage* ***A5.2***

A5.1.1 Bitte geben Sie die Medikamente an, mit denen es behandelt wurde. ❖ *Bei weiteren Medikamenten bitte auf einem Extrablatt auflisten*

1. Medikament

Name: ________________________________

mg: ________________________________

Wie häufig (falls bekannt):

____

2. Medikament

Name: ________________________________

mg: ________________________________

Wie häufig (falls bekannt):

____

3. Medikament

Name: ________________________________

mg: ________________________________

Wie häufig (falls bekannt):

____

7/8 *frühere Version*

A5.2 Wurde/Wird bei Ihrem Kind wegen Heuschnupfen eine Hyposensibilisierungsbehandlung durchgeführt?

☐ 1 Ja

☐ 0 Nein → *Gehen Sie bitte weiter zu Frage* ***A5.3***

A5.2.1 Bitte nennen Sie die Art der Hyposensibilisierung (z.B. Spritzen, Tropfen, Tabletten)

____

____

A5.3 Ist Ihr Kind jemals wegen Heuschnupfen mit sogenannten alternativen Methoden (Homöopathie, Akupunktur, Bioresonanz) behandelt worden?

☐1 Ja ☐0 Nein → *Gehen Sie bitte weiter zu Frage A6*

A5.3.1 Bitte nennen Sie die Art der Behandlung

____

____

A5.3.2 Wie alt war Ihr Kind?

|___|___| [Jahre]

Die folgenden Fragen zu der Haut Ihres Kindes wiederholen sich teilweise mit unterschiedlichem Zeitbezug, um unsere Studienergebnisse mit den Ergebnissen anderer Studien vergleichen zu können.

A6 Hatte Ihr Kind in den letzten 12 Monaten einen juckenden Hautausschlag, der stärker oder schwächer über mindestens 2 Wochen Dauer anhielt?

☐1 Ja ☐0 Nein → *Gehen Sie bitte weiter zu Frage A7*

A6.1	Trat dieser Hautausschlag bei Ihrem Kind in Hautfaltenregionen (Ellenbeugen, Kniekehlen, Hand-oder Fußgelenke, Hals, Augenumgebung) auf?	☐1 Ja<br>☐0 Nein

A7 Hatte Ihr Kind irgendwann einmal einen juckenden Hautausschlag, der stärker oder schwächer über mindestens 6 Monate auftrat?

☐1 Ja ☐0 Nein → *Gehen Sie bitte weiter zu Frage A8*

A7.1	Trat dieser juckende Hautausschlag bei Ihrem Kind auch in den letzten 12 Monaten auf?	☐1 Ja<br>☐0 Nein → *Gehen Sie bitte weiter zu Frage A8*
A7.2	Trat dieser juckende Hautausschlag bei Ihrem Kind irgendwann einmal an einer der folgenden Körperstellen auf: In den Ellenbeugen oder Kniekehlen, an den Hand- oder Fußgelenken, im Gesicht, am Hals?	☐1 Ja<br>☐0 Nein

A8 Hatte Ihr Kind irgendwann einmal Neurodermitis (Atopisches Ekzem, Endogenes Ekzem)?

☐1 Ja ☐0 Nein → *Gehen Sie bitte weiter zu Frage A9*

A8.1	Wann trat die Neurodermitis (Atopisches Ekzem, Endogenes Ekzem) zum ersten Mal auf?	|___|___| [Jahre]

8 | 9 frühere Version

A8.2 Besteht die Erkrankung weiterhin?

☐ $_0$ Nein ☐ $_1$ Ja → *Gehen Sie bitte weiter <u>zu Frage</u>* ***A8.3***

A8.2.1 Seit welchem Alter ist Ihr Kind beschwerdefrei?

|___|___| [Jahre]

A8.3 Ist Ihr Kind in den <u>letzten 12 Monaten</u> wegen Neurodermitis (Atopisches Ekzem, Endogenes Ekzem) behandelt worden?

☐ $_1$ Ja ☐ $_0$ Nein → *Gehen Sie bitte weiter <u>zu Frage</u>* ***A8.4***

9 / 10 *frühere Version*

A8.3.1 Bitte geben Sie die Medikamente und Cremes/Salben an, mit denen es behandelt wurde.

❖ *Bei weiteren Medikamenten bitte auf einem Extrablatt auflisten*

1. Medikament
   Name: ______________________________
   mg: ______________________________
   Wie häufig (falls bekannt):
   ____

2. Medikament
   Name: ______________________________
   mg: ______________________________
   Wie häufig (falls bekannt):
   ____

3. Medikament
   Name: ______________________________
   mg: ______________________________
   Wie häufig (falls bekannt):
   ____

A8.4 Ist Ihr Kind <u>jemals</u> wegen Neurodermitis mit sogenannten alternativen Methoden (Homöopathie, Akupunktur, Bioresonanz) behandelt worden?

☐ $_1$ Ja ☐ $_0$ Nein → *Gehen Sie bitte weiter <u>zu Frage</u>* ***A9***

A8.4.1 Bitte nennen Sie die Art der Behandlung

____

____

10 / 11 *frühere Version*

A8.4.2 Wie alt war Ihr Kind?

|___|___| [Jahre]

A9 Hat ein Arzt bei Ihrem Kind in den vergangenen 4 Jahren eine der folgenden Krankheiten festgestellt?
Wenn ja, geben Sie bitte das Alter des ersten Auftretens an.

	Nein	Ja	im Alter von
Bronchitis	☐0	☐1	|___|___| Jahren
Obstruktive, asthmoide oder spastische Bronchitis	☐0	☐1	|___|___| Jahren<br>Wie oft in den letzten 12 Monaten:<br>|___|___|
Lungenentzündung	☐0	☐1	|___|___| Jahren
Asthma	☐0	☐1	|___|___| Jahren
Mittelohrentzündung	☐0	☐1	|___|___| Jahren
Polypen (Adenoide)	☐0	☐1	|___|___| Jahren
Wurmerkrankungen	☐0	☐1	|___|___| Jahren
Nesselsucht, Urtikaria oder Quincke-Ödem	☐0	☐1	|___|___| Jahren
Nahrungsmittelallergie	☐0	☐1	|___|___| Jahren
Neurodermitis, allergisches oder atopisches Ekzem	☐0	☐1	|___|___| Jahren
Ekzem ohne nähere Angabe	☐0	☐1	|___|___| Jahren
Heuschnupfen	☐0	☐1	|___|___| Jahren
Ganzjährig allergischer Schnupfen, z.B. Hausstaubmilbenallergie	☐0	☐1	|___|___| Jahren
Sonstige chronische Krankheiten (z.B. Herzfehler, Diabetes, Zöliakie):	☐0	☐1	|___|___| Jahren<br>1. Erkrankung:<br>______________________________<br>|___|___| Jahren<br>2. Erkrankung:<br>______________________________<br>|___|___| Jahren<br>3. Erkrankung:<br>______________________________

11
12
frühere Version

A10 War Ihr Kind in den letzten 12 Monaten erkältet?
(Infekte des Nasenrachenraumes, der Bronchien, der Lunge oder der Ohren)

☐1 Ja, ungefähr |___|___| mal ☐0 Nein

A11 Hatte Ihr Kind <u>in den letzten 12 Monaten</u> eine fieberhafte Erkältungskrankheit?

☐$_1$ Ja, ungefähr |___|___| mal ☐$_0$ Nein

12 / 13 *frühere Version*

A12 Hustet Ihr Kind häufig beim Aufstehen oder sonst im Laufe des Tages, ohne erkältet zu sein?

☐$_1$ Ja ☐$_0$ Nein

A13 Erkrankte Ihr Kind <u>in den letzten 12 Monaten</u> an einer eitrigen Mandelentzündung?

☐$_1$ Ja, ungefähr |___|___| mal ☐$_0$ Nein
☐$_2$ Mandeln wurden entfernt im Alter von |___|___| Jahren

A14 Nimmt Ihr Kind regelmäßig Nahrungsergänzungsmittel ein?

☐$_1$ Ja ☐$_0$ Nein ➔ *Gehen Sie bitte weiter <u>zu Frage</u>* ***<u>U1</u>***

A14.1 Bitte geben Sie an, welche Nahrungsergänzungsmittel Ihr Kind einnimmt. ❖ *Bei weiteren Präparaten bitte auf einem Extrablatt auflisten*

1. Nahrungsergänzungsmittel
   Name: ______________________________
   mg: ______________________________
   Wie häufig (falls bekannt):
   ____

2. Nahrungsergänzungsmittel
   Name: ______________________________
   mg: ______________________________
   Wie häufig (falls bekannt):
   ____

3. Nahrungsergänzungsmittel
   Name: ______________________________
   mg: ______________________________
   Wie häufig (falls bekannt):
   ____

CHARITÉ hhu Heinrich Heine Universität Düsseldorf

13 / 14 *frühere Version*

Aktivitäten und Umgebung

U1 Während einer normalen Woche (außerhalb eines Corona-Lockdowns): wie viele Stunden pro Tag (24 Stunden) verbringt Ihr Kind vor einem Bildschirm (Fernseher, Computer, Smartphone, Spielekonsole)?

Sommer	Winter	
☐1	☐1	Weniger als 1 Stunde pro Tag
☐2	☐2	1 bis 2 Stunden pro Tag
☐3	☐3	3 bis 4 Stunden pro Tag
☐4	☐4	5 Stunden oder mehr

U2 Ist Ihr Kind aktives Mitglied in einem Sportverein /Sport-AG?

☐1 Ja ☐0 Nein → *Gehen Sie bitte weiter zu Frage* ***U3***

U2.1 Welche Sportart/en betreibt Ihr Kind?

1. Sportart:

____

2. Sportart:

____

3. Sportart:

____

4. Sportart:

____

5. Sportart:

____

6. Sportart:

____

14 / 15 *frühere Version*

U3 Wie viele Stunden in einer normalen Woche (7 Tage, nicht im Lockdown) ist Ihr Kind körperlich aktiv?

	Sommer	Winter
leichte körperliche Aktivität (ohne Schwitzen, normale Atmung, z.B. Gehen)	|___|___|	|___|___| Stunden / Woche
mittelschwere körperliche Aktivität (etwas Schwitzen, leicht erhöhte Atmung z.B. Radfahren, Schwimmen, Skaten)	|___|___|	|___|___| Stunden / Woche
schwere körperliche Aktivität (viel Schwitzen, schnelle Atmung, z.B. Ballspiele, Training)	|___|___|	|___|___| Stunden / Woche

U4 Halten/Hielten Sie seit der Geburt Ihres Kindes jemals Haustiere in der Wohnung?

☐1 Ja ☐0 Nein → *Gehen Sie bitte weiter zu Frage* ***U5***

U4.1 Welche Haustiere hielten Sie im letzten Jahr?

	Anzahl
☐ Hund	|___|___|
☐ Katze	|___|___|
☐ Meerschweinchen	|___|___|
☐ Hamster, Kaninchen	|___|___|
☐ Ziervögel	|___|___|
☐ Sonstige	|___|___|

U5 Hat Ihr Kind außerhalb der Wohnung regelmäßig Kontakt zu Tieren?

☐$_1$ Ja ☐$_0$ Nein → *Gehen Sie bitte weiter zu Frage* ***U6***

U5.1 Zu welchen Tieren?

☐$_1$ Hund ☐$_3$ Pferd

☐$_2$ Katze ☐$_4$ Sonstige

15/16

frühere Version

U6 Wie oft wurde während des letzten Jahres in Ihrer Wohnung geraucht?

☐$_0$ Nie → *Gehen Sie bitte weiter zu Frage* ***U7***

☐$_1$ Gelegentlich

☐$_2$ Mindestens 1 Mal pro Woche

☐$_3$ Täglich oder fast täglich

U6.1 Wie viele Zigaretten pro Tag wurden während des letzten Jahres in Ihrer Wohnung durchschnittlich geraucht?

Mutter/Erziehungsberechtigte |___|___| Anzahl

Vater/Erziehungsberechtigter |___|___| Anzahl

Andere Person |___|___| Anzahl

U7 Wurde während des letzten Jahres bei Ihnen auf dem Balkon oder der Terrasse geraucht?

☐$_1$ Ja ☐$_0$ Nein

U8 Wie oft war Ihr Kind während des letzten Jahres außerhalb der Wohnung Tabakrauch ausgesetzt?

☐$_0$ Nie ☐$_2$ Mindestens 1 Mal pro Woche

☐$_1$ Gelegentlich ☐$_3$ Täglich oder fast täglich

U9 Wie viele Quadratmeter misst Ihre Wohnung etwa insgesamt?

|___|___|___| m^2

U10 Wie viele Zimmer (ohne Küche, Bad) hat Ihre Wohnung

|___|___| Anzahl

U11 Würden Sie Ihre Wohnung als feucht bezeichnen?

☐$_1$ Ja ☐$_0$ Nein

U12 Gibt es Schimmel- oder Stockflecken in folgenden Räumen (außer auf Nahrungsmitteln)?

❖ *Mehrfachnennungen möglich*

☐ Schlafzimmer/Kinderzimmer des Kindes ☐ Keller

16 ☐ Übrige Wohnung ☐ Nirgends

17 *frühere Version*

U13 Wie wird Ihre Wohnung überwiegend beheizt?

☐$_2$ Einzelofen/Brenner in der Wohnung

☐$_1$ Ofen/Brenner außerhalb der Wohnung (Zentralwärme, Ofen im Keller)

➔ *Gehen Sie bitte weiter <u>zu Frage **U14**</u>*

U13.1 Wenn Sie einen Einzelofen/Brenner in der Wohnung haben, welches Heizmaterial verwenden Sie?

☐$_1$ Gas ☐$_3$ Vorwiegend Holz

☐$_2$ Vorwiegend Kohle ☐$_4$ Öl

☐$_5$ andere (z.B. Elektroheizung, Nachtspeicher)

U14 Benutzen Sie einen Gasherd zum Kochen?

☐$_1$ Ja ☐$_0$ Nein

U15 Wie viele Personen schlafen mit Ihrem Kind zusammen in einem Raum (dieses Kind nicht mitgezählt)?

|___|___| Anzahl Personen

U16 Auf welcher Etage befindet sich das Zimmer des Kindes?

|___|___| Etage

U17 Zu welcher Straße liegt das Zimmer, in dem Ihr Kind schläft?

❖ *Wenn das Zimmer zu mehreren Straßen liegt, wählen Sie bitte <u>**die lauteste**</u> Straße.*

☐$_1$ zur Autobahn ☐$_4$ zum Innenhof

☐$_2$ zur Hauptverkehrsstraße ☐$_5$ zur Grünanlage/ zum Garten (keine Straße)

☐$_3$ zur Nebenstraße

U18 Welche Beschaffenheit haben die Fenster in dem Zimmer, in dem Ihr Kind schläft?

☐$_1$ Schallschutzfenster ☐$_3$ Einfachfenster

17 ☐$_2$ Doppelfenster oder Isolierverglasung ☐$_4$ Sonstige Fenster

18 *frühere Version*

U19 An welcher Straße wohnen Sie?

☐$_1$ Spielstraße, Sackgasse ☐$_3$ Nebenstraße ohne Tempo 30-Limit

☐$_2$ Nebenstraße mit Tempo 30-Limit ☐$_4$ Hauptstraße

U20 Wie würden Sie den Verkehr in der Straße, in der Sie wohnen, beschreiben? ❖ *Mehrfachnennungen möglich*

☐ Anwohnerverkehr (in Wohngebieten) ☐ Berufsverkehr

☐ Durchgangsverkehr

U21 Kommt es in den Hauptverkehrszeiten regelmäßig zur Staubildung?

☐₁ Ja ☐₀ Nein

U22 Wie stark fühlen Sie sich zu Hause durch Straßen- und Flugzeuglärm gestört, wenn Sie das Fenster geöffnet haben? ❖ *Kreuzen Sie bitte das entsprechende Kästchen für den Grad der Störung an.*

Verkehrslärm:		☐	☐	☐	☐	☐	☐	☐	☐	☐	☐	☐
stört kein bisschen	0	1	2	3	4	5	6	7	8	9	10	stört unerträglich
Flugzeuglärm:		☐	☐	☐	☐	☐	☐	☐	☐	☐	☐	☐
stört kein bisschen	0	1	2	3	4	5	6	7	8	9	10	stört unerträglich

U23 Wie viele Stunden hält sich Ihr Kind durchschnittlich draußen auf?

Im Sommer |___|___| Stunden/Tag Im Winter |___|___| Stunden/Tag

U24 Sind Sie eine alleinerziehende Mutter oder ein alleinerziehender Vater?

☐₁ Ja ☐₀ Nein

U25 Sind Sie zurzeit erwerbstätig?
❖ *Bitte sofern möglich für beide hier angegeben Personen ausfüllen.*

	Vollerwerb-stätig	Halbtags	Weniger als halbtags	Gar nicht
Mutter/ Erziehungsberechtigte	☐₁	☐₂	☐₃	☐₄
Vater/ Erziehungsberechtigter	☐₁	☐₂	☐₃	☐₄

18
19

U26 Welchen höchsten allgemeinen Schulabschluss haben Sie?
❖ *Bitte sofern möglich für beide hier angegeben Personen ausfüllen.*

frühere Version

Mut-ter/Erziehungsbere chtigte	Vater/ Erziehungs-berechtigter	
☐₁	☐₁	Schulabgang ohne Abschluss
☐₂	☐₂	Hauptschulabschluss / Volksschulabschluss
☐₃	☐₃	Polytechnische Oberschule 10. Klasse (vor 1965: 8. Klasse)
☐₄	☐₄	Fachhochschulreife / fachgebundene Hochschulreife / Fachoberschule
☐₅	☐₅	allgemeine Hochschulreife (Abitur, EOS, Berufsausbildung mit Abitur)
☐₆	☐₆	anderer Schulabschluss: ________________
☐₇	☐₇	noch in Schulausbildung, Art der Schule: ________________

❖ **Bitte wenden!**

U27 Wie viele Geschwister hat Ihr Kind?

|___|___| Anzahl

U28 Wie viele leibliche Geschwister (keine Halbgeschwister) hat Ihr Kind?

|___|___| Anzahl

19/20 *frühere Version*

U29 Hatte eines der leiblichen Geschwister jemals eine der folgenden Krankheiten?

Geschwister	Asthma	Heuschnupfen	Neurodermitis, atopisches Ekzem
1. |___|___|___|___| [Geburtsjahr] männlich ☐ weiblich ☐	☐1 Ja ☐0 Nein	☐1 Ja ☐0 Nein	☐1 Ja ☐0 Nein
2. |___|___|___|___| [Geburtsjahr] männlich ☐ weiblich ☐	☐1 Ja ☐0 Nein	☐1 Ja ☐0 Nein	☐1 Ja ☐0 Nein
3. |___|___|___|___| [Geburtsjahr] männlich ☐ weiblich ☐	☐1 Ja ☐0 Nein	☐1 Ja ☐0 Nein	☐1 Ja ☐0 Nein
4. |___|___|___|___| [Geburtsjahr] männlich ☐ weiblich ☐	☐1 Ja ☐0 Nein	☐1 Ja ☐0 Nein	☐1 Ja ☐0 Nein
5. |___|___|___|___| [Geburtsjahr] männlich ☐ weiblich ☐	☐1 Ja ☐0 Nein	☐1 Ja ☐0 Nein	☐1 Ja ☐0 Nein
6. |___|___|___|___| [Geburtsjahr] männlich ☐ weiblich ☐	☐1 Ja ☐0 Nein	☐1 Ja ☐0 Nein	☐1 Ja ☐0 Nein

❖ *Bei weiteren Geschwistern bitte auf einem Extrablatt auflisten*

Herzlichen Dank für Ihre Mitarbeit!
