# Supplementary material for: The Berlin-Brandenburg Air Study—A Methodological Study Paper of a Natural Experiment Investigating Health Effects Related to Changes in Airport-Related Exposures
Source: Int J Public Health. 2023 Nov 17;68:1606096. doi: 10.3389/ijph.2023.1606096 (PMC10689260; doi:10.3389/ijph.2023.1606096)
Supplement: Supplementary file 2 [file DataSheet4.pdf]

Deckblatt Kid-KINDL^{R}

[wird vom Studienpersonal ausgefüllt]

ID-Nummer: |___|___|___|___|___|

Untersucher-Nr: |___|___|

Datum: |___|___|.|___|___|.|___|___|___|___|[TT.MM.JJJJ]

Uhrzeit: |___|___|:|___|___| [hh:mm]

Fragebogen für Kinder

Kid-KINDL[R]

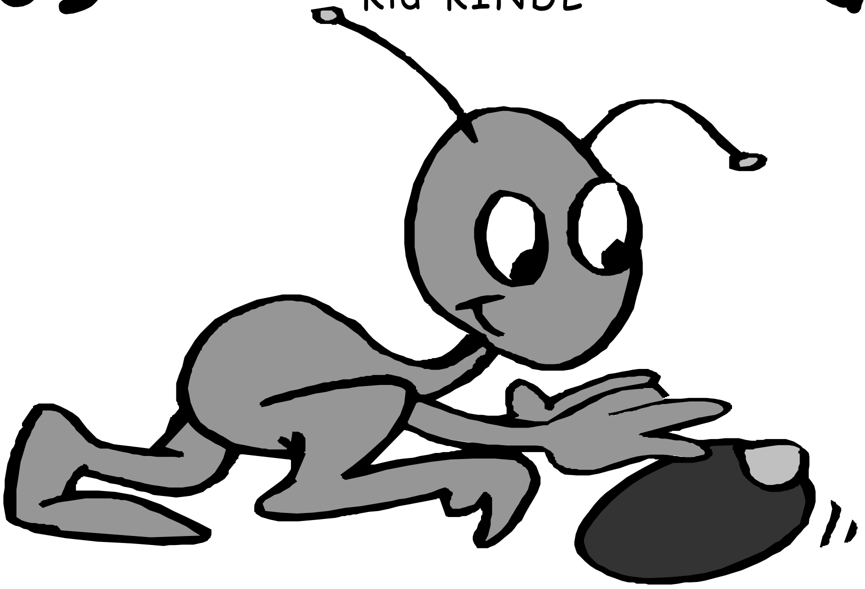

Hallo,

wir möchten gerne wissen, wie es dir zur Zeit geht. Dazu haben wir uns einige Fragen ausgedacht und bitten dich um deine Antwort.

- ➪ Lies bitte jede Frage durch,
- ➪ überlege, wie es in der letzten Woche war,
- ➪ kreuze **in jeder Zeile** die Antwort an, die am besten zu dir passt.

Es gibt keine richtigen oder falschen Antworten.
Wichtig ist uns deine Meinung.

**Ein Beispiel:** ✎	nie	selten	manch-mal	oft	Immer
In der letzten Woche habe ich gerne Musik gehört	☐	☐	☐	☒	☐

Bogen ausgefüllt am:

____

Tag/Monat/Jahr

© Kid-KINDL[R] / Kinderversion / Ravens-Sieberer & Bullinger / 2000

<u>Bitte sage uns zunächst etwas zu dir. Kreuze an oder trage ein !</u>

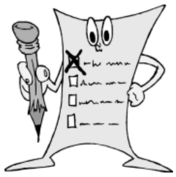

Ich bin ein ☐ Mädchen ☐ Junge

Ich bin ______ Jahre alt

Wieviele Geschwister hast du? ☐ 0 ☐ 1 ☐ 2 ☐ 3 ☐ 4 ☐ 5 ☐ über 5

Welche Schule besuchst du? ☐ Grundschule ☐ Hauptschule ☐ Realschule ☐ Gesamtschule ☐ Gymnasium ☐ Sonderschule ☐ privater Unterricht

1. Zuerst möchten wir etwas über deinen Körper wissen, ...

	In der letzten Woche ...	nie	selten	manch-mal	oft	immer
1.	... habe ich mich krank gefühlt	☐	☐	☐	☐	☐
2.	... hatte ich Kopfschmerzen oder Bauchschmerzen	☐	☐	☐	☐	☐
3.	... war ich müde und schlapp	☐	☐	☐	☐	☐
4.	... hatte ich viel Kraft und Ausdauer	☐	☐	☐	☐	☐

2. ... dann etwas darüber, wie du dich fühlst ...

	In der letzten Woche ...	nie	selten	manch-mal	oft	immer
1.	... habe ich viel gelacht und Spaß gehabt	☐	☐	☐	☐	☐
2.	... war mir langweilig	☐	☐	☐	☐	☐
3.	... habe ich mich allein gefühlt	☐	☐	☐	☐	☐
4.	... habe ich Angst gehabt	☐	☐	☐	☐	☐

3. ... und was du selbst von dir hältst.

	In der letzten Woche ...	nie	selten	manch-mal	oft	immer
1.	... war ich stolz auf mich	☐	☐	☐	☐	☐
2.	... fand ich mich gut	☐	☐	☐	☐	☐
3.	... mochte ich mich selbst leiden	☐	☐	☐	☐	☐
4.	... hatte ich viele gute Ideen	☐	☐	☐	☐	☐

4. In den nächsten Fragen geht es um deine Familie ...

	In der letzten Woche ...	nie	selten	manch-mal	oft	immer
1.	... habe ich mich gut mit meinen Eltern verstanden	☐	☐	☐	☐	☐
2.	... habe ich mich zu Hause wohl gefühlt	☐	☐	☐	☐	☐
3.	... hatten wir schlimmen Streit zu Hause	☐	☐	☐	☐	☐
4.	... haben mir meine Eltern Sachen verboten	☐	☐	☐	☐	☐

5. ... und danach um Freunde.

	In der letzten Woche ...	nie	selten	manch-mal	oft	immer
1.	... habe ich mit Freunden gespielt	☐	☐	☐	☐	☐
2.	... mochten mich die anderen Kinder	☐	☐	☐	☐	☐
3.	... habe ich mich mit meinen Freunden gut verstanden	☐	☐	☐	☐	☐
4.	... hatte ich das Gefühl, dass ich anders bin als die anderen	☐	☐	☐	☐	☐

6. Nun möchten wir noch etwas über die Schule wissen.

	In der letzten Woche, in der ich in der Schule war ...	nie	selten	manch-mal	oft	immer
1.	... habe ich die Schulaufgaben gut geschafft	☐	☐	☐	☐	☐
2.	... hat mir der Unterricht Spaß gemacht	☐	☐	☐	☐	☐
3.	... habe ich mir Sorgen um meine Zukunft gemacht	☐	☐	☐	☐	☐
4.	... habe ich Angst vor schlechten Noten gehabt	☐	☐	☐	☐	☐

VIELEN DANK FÜR DEINE MITARBEIT!
