# Supplementary figures and images for: The Berlin-Brandenburg Air Study—A Methodological Study Paper of a Natural Experiment Investigating Health Effects Related to Changes in Airport-Related Exposures
Source: Int J Public Health. 2023 Nov 17;68:1606096. doi: 10.3389/ijph.2023.1606096 (PMC10689260; doi:10.3389/ijph.2023.1606096)

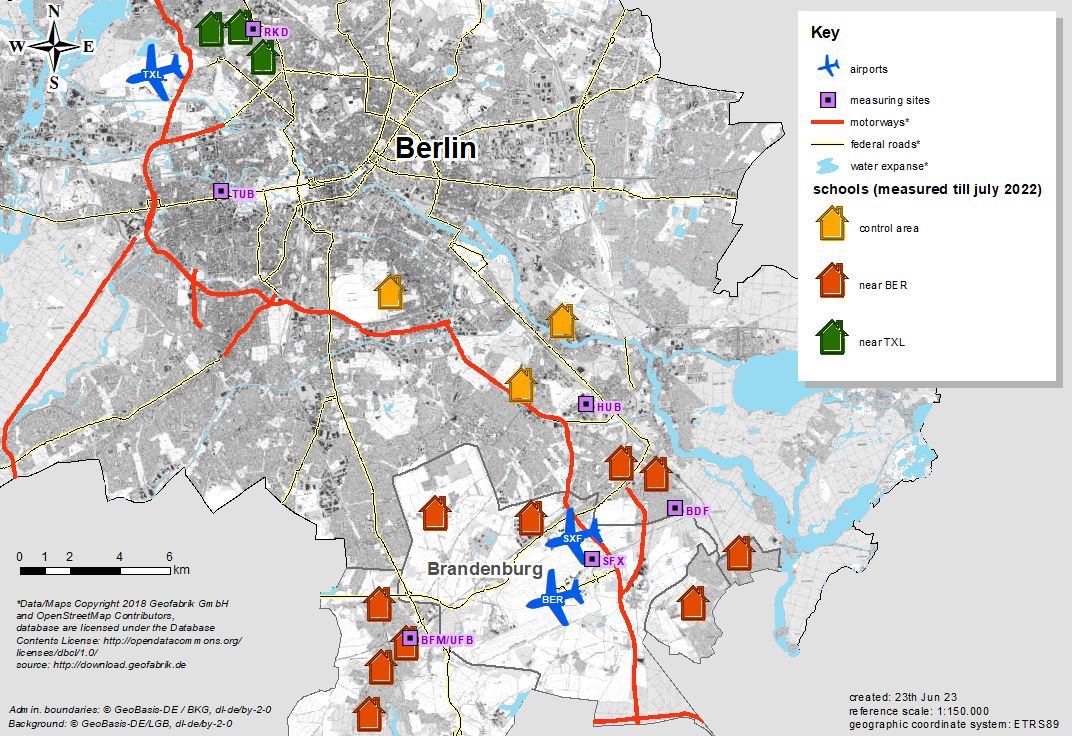

Supplement: Supplementary file 3 [file Image1.JPEG]
