# Supplementary material for: The Berlin-Brandenburg Air Study—A Methodological Study Paper of a Natural Experiment Investigating Health Effects Related to Changes in Airport-Related Exposures
Source: Int J Public Health. 2023 Nov 17;68:1606096. doi: 10.3389/ijph.2023.1606096 (PMC10689260; doi:10.3389/ijph.2023.1606096)
Supplement: Supplementary file 4 [file DataSheet3.pdf]

Abfrage akuter Beschwerden

ID-Nummer: |___|___|___|___|___|

Untersucher-Nr: |___|___|

Datum: |___|___|.|___|___|.|___|___|___|___|[TT.MM.JJJJ]

Uhrzeit: |___|___|:|___|___| [hh:mm]

Abkürzung Schule: ________________

Hinweis Studienpersonal: bitte Kürzel aus Liste der Schulen verwenden und gut lesbar schreiben!

B1	Fühlst Du Dich heute fit genug und hast Lust an dieser Studie teilzunehmen?<br><br>(Nachdem das Kind mit „ja“ geantwortet hat folgt eine positive Bestätigung von uns)	☐ 1 Ja<br>☐ 0 Nein<br><br>Falls „Nein“, warum nicht?<br>________________<br>________________
B2	Bist du heute sehr müde?	☐ 1 Ja<br>☐ 0 Nein<br>☐ 8 Keine Angabe *(nicht vorlesen!)*<br>☐ 9 Weiß nicht *(nicht vorlesen!)*
B3	Bist Du heute sehr erkältet mit laufender Nase und Husten? Oder warst du in den letzten Tagen so erkältet, dass du nicht zur Schule oder zum Sport gehen durftest?	☐ 1 Ja<br>☐ 0 Nein<br>☐ 8 Keine Angabe *(nicht vorlesen!)*<br>☐ 9 Weiß nicht *(nicht vorlesen!)*

B4	Hast Du heute oder hattest Du in den letzten Tagen eine andere Erkrankung?	☐ 1 Ja<br>☐ 0 Nein<br>☐ 8 Keine Angabe *(nicht vorlesen!)*<br>☐ 9 Weiß nicht *(nicht vorlesen!)*<br><br>Falls „Ja“, welche?<br><br>______________________________<br><br>______________________________
B5	Hast Du heute oder hattest Du in den letzten Tagen eine Allergie?	☐ 1 Ja<br>☐ 0 Nein<br>☐ 8 Keine Angabe *(nicht vorlesen!)*<br>☐ 9 Weiß nicht *(nicht vorlesen!)*
B6	All diese Fragen stellen wir auch älteren Kindern und Jugendlichen, deshalb auch diese Frage:<br><br>Hast Du heute oder in den letzten Tagen geraucht?	☐ 1 Ja<br>☐ 0 Nein<br>☐ 8 Keine Angabe *(nicht vorlesen!)*<br>☐ 9 Weiß nicht *(nicht vorlesen!)*
B7	Hat jemand in Deiner Umgebung heute oder in den letzten Tagen geraucht, so dass Du den Rauch eingeatmet hast?	☐ 1 Ja<br>☐ 0 Nein<br>☐ 8 Keine Angabe *(nicht vorlesen!)*<br>☐ 9 Weiß nicht *(nicht vorlesen!)*
B8	Hast Du heute oder gestern ganz viel Sport gemacht? (z.B. > 3 Stunden am Tag)?	☐ 1 Ja<br>☐ 0 Nein<br>☐ 8 Keine Angabe *(nicht vorlesen!)*<br>☐ 9 Weiß nicht *(nicht vorlesen!)*

B9	Hast Du heute oder hattest Du in den letzten Tagen Heuschnupfen?	☐ 1 Ja<br>☐ 0 Nein<br>☐ 8 Keine Angabe *(nicht vorlesen!)*<br>☐ 9 Weiß nicht *(nicht vorlesen!)*
B10	Hast Du heute oder hattest Du in den letzten Tagen einen juckenden Ausschlag?	☐ 1 Ja<br>☐ 0 Nein<br>☐ 8 Keine Angabe *(nicht vorlesen!)*<br>☐ 9 Weiß nicht *(nicht vorlesen!)*
B11	Hast Du heute oder hattest Du in den letzten Tagen rote und tränende Augen?	☐ 1 Ja<br>☐ 0 Nein<br>☐ 8 Keine Angabe *(nicht vorlesen!)*<br>☐ 9 Weiß nicht *(nicht vorlesen!)*
B12	Hast Du heute oder hattest Du in den letzten Tagen Schwierigkeiten beim Atmen?	☐ 1 Ja<br>☐ 0 Nein<br>☐ 8 Keine Angabe *(nicht vorlesen!)*<br>☐ 9 Weiß nicht *(nicht vorlesen!)*

B13	Hast Du in den letzten 7 Tagen mehrmals Medikamente eingenommen, z. Bsp. Tabletten, Cremes, Salben, Tropfen oder Spray´s ?<br><br>Wenn ja, welche und wofür?	☐ 1 Ja<br>☐ 0 Nein<br>☐ 8 Keine Angabe *(nicht vorlesen!)*<br>☐ 9 Weiß nicht *(nicht vorlesen!)*<br><br>Falls „Ja“:<br>1. Medikament<br>Name: ______________________<br>Wofür: ______________________<br>2. Medikament<br>Name: ______________________<br>Wofür: ______________________<br>3. Medikament<br>Name: ______________________<br>Wofür: ______________________<br><br>*Weitere Medikamente auf Extrablatt notieren*
B14	Wann bist Du heute zu Hause losgegangen?<br><br>Bei ungefähren Angaben den Mittelwert angeben.	|___|___|:|___|___| Uhr [hh:mm]<br>☐ 1 keine Angabe *(nicht vorlesen!)*<br>☐ 1 Weiß nicht *(nicht vorlesen!)*
B15	Wie bist Du heute zur Schule gekommen?	☐ 1 Auto<br>☐ 2 Bus & Bahn<br>☐ 3 Zu Fuß<br>☐ 4 Mit dem Fahrrad oder Roller<br>☐ 8 keine Angabe *(nicht vorlesen!)*
B16	Wie lange warst Du heute unterwegs? Bei ungefähren Angaben den Mittelwert angeben.	|___|___| Stunden |___|___| Minuten<br>☐ 1 keine Angabe *(nicht vorlesen!)*<br>☐ 1 Weiß nicht *(nicht vorlesen!)*
