# Supplementary material for: The Berlin-Brandenburg Air Study—A Methodological Study Paper of a Natural Experiment Investigating Health Effects Related to Changes in Airport-Related Exposures
Source: Int J Public Health. 2023 Nov 17;68:1606096. doi: 10.3389/ijph.2023.1606096 (PMC10689260; doi:10.3389/ijph.2023.1606096)
Supplement: Supplementary file 5 [file DataSheet1.pdf]

Supplement

Recruitment of schools

Schools and their affiliated after-school centers were recruited for the study mainly from three areas: near the Tegel Airport (TLX), near the Berlin-Brandenburg Airport (BER), and from control areas (CA) away from both airports (Supplementary Figure S1 and Table S1) (insert Figure S1 and Table S1). The schools near TLX and BER were located in 4 km and 6 km away from the nearest airport in average. The schools in the control areas were located in 17 km away from TLX and in 11 km away from BER in average (Supplementary Figure S1 and Table S1).

We submitted an application to the Berlin Senate Department for Education, Youth and Family to conduct the study in the schools of Berlin and received approval. All eligible schools were contacted by telephone via the respective school administration. The contents, goals and the design of the BEAR study were explained to the headmasters. If the headmasters decided to participate in the study, this decision was discussed in the school conference, consisting of school management, teachers, parents and elementary school children.

Unfortunately, the Ministry of Education, Youth and Sports of the State of Brandenburg disagreed the implementation of the BEAR study in elementary schools in Brandenburg. So we asked the mayors of the Brandenburg municipalities (adjacent to BER airport), who are responsible for the after-school care in the schools in the federal state of Brandenburg, for participation in the BEAR study. After agreement, we contacted the after-school care management to plan the organizational process. The investigations for the BEAR study are carried out in the after-school care in the municipalities of Blankenfelde-Mahlow, Schönefeld, Schulzendorf and Eichwalde.

TABLE S1. Participating schools in Berlin and Brandenburg and distance to the airports Berlin-Brandenburg (BER) and Berlin-Tegel (TLX) (Berlin-Brandenburg Air Study, Germany 2020-2024)

Study area	Elementary school (district of Berlin/ municipality of Brandenburg)	Distance to BER [km]	Distance to TLX [km]
Control Area (CA)	CA-1 (Berlin-Johannisthal)	8.6	18.8
	CA-2 (Berlin-Oberschöneweide)	11.0	18.9
	CA-3 (Berlin-Neukölln)	13.8	12.4
TLX	TLX-1 (Berlin-Reinickendorf)	25.9	4.1
	TLX-2 (Berlin-Reinickendorf)	26.4	3.1
	TLX-3 (Berlin-Reinickendorf)	24.7	4.3
SFX/BER	BER-1 (Berlin-Bohnsdorf)	6.6	25.2
	BER-2 (Berlin-Altglienicke)	6.2	23.8
	BER-3 (Schulzendorf, Brandenburg)	5.6	29.4
	BER-4 (Eichwalde, Brandenburg)	7.8	29.7
	BER-5 (Schönefeld, Brandenburg)	3.3	22.8
	BER-6 (Schönefeld, Brandenburg)	5.7	20.3
	BER-7 (Blankenfelde-Mahlow, Brandenburg)	7.2	24.8
	BER-8 (Blankenfelde-Mahlow, Brandenburg)	8.5	26.4
	BER-9 (Blankenfelde-Mahlow, Brandenburg)	5.6	24.3
	BER-10 (Blankenfelde-Mahlow, Brandenburg)	6.8	22.5

FIGURE S1. Map showing schools in the vicinity of Tegel Airport (TXL) (green), Schönefeld and Berlin-Brandenburg Airport (SFX/BER) (red) and in control areas (CA) (yellow) (as of July 2022). Airports are shown with a blue airplane and the monitoring stations are marked in purple (Berlin-Brandenburg Air Study, Germany 2020-2024).
